# Preoperative Nomogram and Risk Calculator for Postoperative Hypoxemia and Related Clinical Outcomes Following Stanford Type A Acute Aortic Dissection Surgery

**DOI:** 10.3389/fcvm.2022.851447

**Published:** 2022-04-25

**Authors:** Weiyong Sheng, Sheng Le, Yu Song, Yifan Du, Jia Wu, Chuanbin Tang, Hongfei Wang, Xing Chen, Su Wang, Jingjing Luo, Rui Li, Jiahong Xia, Xiaofan Huang, Ping Ye, Long Wu, Xinling Du, Dashuai Wang

**Affiliations:** ^1^Department of Emergency General Surgery, Union Hospital, Tongji Medical College, Huazhong University of Science and Technology, Wuhan, China; ^2^Department of Cardiovascular Surgery, Union Hospital, Tongji Medical College, Huazhong University of Science and Technology, Wuhan, China; ^3^Department of Thoracic Surgery, Zhongnan Hospital of Wuhan University, Wuhan University, Wuhan, China; ^4^Key Laboratory for Molecular Diagnosis of Hubei Province, Tongji Medical College, The Central Hospital of Wuhan, Huazhong University of Science and Technology, Wuhan, China; ^5^Department of Emergency Medicine, Union Hospital, Tongji Medical College, Huazhong University of Science and Technology, Wuhan, China; ^6^Department of Cardiology, Union Hospital, Tongji Medical College, Huazhong University of Science and Technology, Wuhan, China; ^7^Department of Cardiovascular Surgery, The First Affiliated Hospital of Zhengzhou University, Zhengzhou, China

**Keywords:** hypoxemia, Stanford type A aortic dissection, risk factor, prediction model, nomogram

## Abstract

**Background:**

Hypoxemia is a common complication after Stanford type A acute aortic dissection surgery (AADS), however, few studies about hypoxemia after AADS exist. The aims of this study were to identify independent risk factors for hypoxemia after AADS and to clarify its association with clinical outcomes.

**Methods:**

Patients undergoing AADS from 2016 to 2019 in our hospital were identified and used as a training set. Preoperative variables were first screened by univariate analysis and then entered into a multivariate logistic regression analysis to identify independent risk factors. A nomogram and an online risk calculator were constructed based on the logistic model to facilitate clinical practice and was externally validated in an independent dataset.

**Results:**

Severe hypoxemia developed in 119 of the 492 included patients (24.2%) and poorer clinical outcomes were observed in these patients. Five independent risk factors for severe hypoxemia after AADS were identified by multivariate analysis, including older age, smoking history, renal insufficiency, higher body mass index, and white blood cell count. The model showed good calibration, discrimination, and clinical utility in the training set, and was well validated in the validation set. Risk stratification was performed and three risk groups were defined as low, medium, and high risk groups. Hypertension was identified as an independent risk factor for moderate hypoxemia besides the five predictors mentioned above, and renal insufficiency was not significant for mild hypoxemia by multivariate analysis. In addition, although frozen elephant trunk was associated with increased risk of postoperative hypoxemia in the univariate analysis, frozen elephant trunk was also not identified as an independent risk factor for postoperative hypoxemia in the multivariate analysis.

**Conclusion:**

Hypoxemia was frequent following AADS, related to poorer clinical outcomes. Predictors were identified and a nomogram as well as an online risk calculator predicting severe hypoxemia after AADS was developed and validated, which may be helpful for risk estimation and perioperative management.

## Introduction

Stanford type A acute aortic dissection is known as a lethal cardiovascular emergency, associated with high disability and mortality (1). Prompt surgical interventions remain the most important treatment despite of considerable improvements have been achieved in diagnostic techniques and medical options over the past few decades (2). Frustratingly, the circumstances of the overall survival after Stanford type A acute aortic dissection surgery (AADS) is hardly optimistic and a high percentage of patients may develop multiple postoperative complications (1).

Postoperative hypoxemia is one of the most common complications after cardiovascular surgery, related to increased risk of morbidity and mortality (3–5). The prevalence of hypoxemia varies widely in the literature due to different definitions and surgical populations sampled in different studies (3, 5–7). Several studies aimed to identify risk factors for the development of hypoxemia after cardiac surgery have been conducted and some predictors have been reported such as smoking history and obesity (3, 5–10). However, only very few studies were carried out in patients undergoing AADS and without exception those studies were performed on small sample sizes. Furthermore, most of those studies included preoperative, intraoperative, and postoperative variables, which may limit the clinical utility of early prediction. Moreover, none of those previous studies developed or externally validated a reasonable visual model such as risk score and risk calculator, which may facilitate clinical application. In addition, no previous studies have systematically investigated the predictors, respectively, for mild, moderate, and severe postoperative hypoxemia. Therefore, our understanding of the risk factors for hypoxemia after AADS is still limited, and the construction and validation of an authentic clinical risk prediction model is still urgently needed. Another noteworthy point is that previous studies only reported the relationship between hypoxemia and clinical outcomes with the results of univariate analysis, which may be largely influenced by confounding factors. Nonetheless, no previous studies have deeply explored the relationship between postoperative hypoxemia and clinical outcomes through multivariate analysis or propensity score matching analysis in patients undergoing AADS.

The aims of this study were first to identify independent risk factors for the development of severe, moderate, and mild hypoxemia, respectively, in patients undergoing AADS and develop risk prediction models; and second to deeply explore the relationship between hypoxemia and clinical outcomes by univariate and propensity score matching analysis.

## Materials and Methods

### Ethics Statement

This study was conducted in accordance with ethical statement of the Declaration of Helsinki. The Ethics Committee of Tongji Medical College of Huazhong University of Science and Technology (IORG No. IORG0003571) approved this study. Written informed consent was waived because of its observational, retrospective nature.

### Study Population and Data Extraction

Consecutive adult patients (age ≥18 years) who underwent AADS in a single cardiovascular center from January 2016 to December 2019 were enrolled. Patients who died intraoperatively were excluded from this study. Clinical data were collected using the hospital’s electronic medical records management system. Preoperative variables incorporated in this study were as follows: demographic variables included sex, age, height, weight, body mass index, smoking, and drinking history; underlying conditions included diabetes mellitus, hypertension, chronic bronchitis, pulmonary emphysema, peripheral vascular disease, cerebrovascular disease, renal insufficiency, gastrointestinal tract disease, atrial fibrillation, cardiac surgery history, general surgical history, New York Heart Association class, pulmonary artery hypertension, pericardial effusion, left ventricular ejection fraction, diameter of the left atrium, left ventricle, right atrium, and right ventricle; laboratory blood tests included white blood cell count, red blood cell count, hemoglobin, platelet count, serum creatinine, serum albumin, and serum globulin. Operative variables included combined surgical types, aortic root surgery, and frozen elephant trunk implantation.

In addition to the data obtained from our hospital, we extracted some clinical data from the MIMIC-IV database^1^ to externally validate the risk prediction model for severe hypoxemia. MIMIC-IV database is a large, longitudinal database that incorporates critical care data at the Beth Israel Deaconess Medical Center between 2008 and 2019. Patient identifiers were removed in this database to strictly protect patient confidentiality. The database is publicly available and allows for data sharing only after passing the Collaborative Institutional Training Initiative examination. One of the authors who had access to MIMIC-IV in our study group specialized in data extraction from this database. Following diagnostic codes of the International Classification of Diseases editions (ICD-9 and ICD-10), the patients diagnosed with “thoracic aortic dissection” and treated with surgical interventions were extracted from the MIMIC-IV database.

### Endpoints and Definitions

The primary endpoint of this study was hypoxemia after AADS. The arterial oxygen tension–inspired oxygen concentration (PaO_2_–FiO_2_) ratios were calculated for the perioperative period. The Berlin definition has been widely accepted for acute respiratory distress syndrome which proposes three categories of hypoxemia on the basis of the degree of the condition. In the present study, according to the diagnostic criteria of the Berlin definition, we defined severe hypoxemia as PaO_2_/FiO_2_ ≤ 100 mmHg, moderate hypoxemia as 100 mmHg < PaO_2_/FiO_2_ ≤ 200 mmHg, and mild hypoxemia as 200 mmHg < PaO_2_/FiO_2_ ≤ 300 mmHg. Afterward, all patients were divided into one of the categories based on the worst values of the recorded PaO_2_/FiO_2_ ratios within the first 24 h postoperatively.

The secondary endpoints were postoperative pneumonia, reintubation, tracheostomy, readmission to intensive care unit (ICU), in-hospital mortality, the lengths of mechanical ventilation, ICU stay, and hospital stay.

### Statistical Analysis

Statistical analyses were performed using SPSS (IBM SPSS Statistics 26.0, SPSS Inc., Chicago, IL, United States) and R software (version 4.0.5^2^). *P*-values less than 0.05 (two-tailed) were considered statistically significant.

The Kolmogorov–Smirnov test was used to evaluate whether continuous variables were distributed normally. Continuous variables were expressed as means ± standard deviations when normally distributed and as medians (interquartile ranges) when skewed. Categorical variables were expressed as counts (percentages). A multiple imputation approach was used to handle with missing data. Univariate analysis was first conducted to screen potential risk factors. Normally distributed continuous variables with homogeneous variance were compared using Student’s *t*-test and otherwise using Mann–Whitney *U*-test. Categorical variables were compared by Chi-square test or Fisher’s exact test. Variables screened by univariate analysis were then entered into a forward stepwise multivariate logistic regression analysis procedure to identify significant risk factors. The odds ratio (OR) was calculated with 95% confidence interval (CI). A nomogram on the basis of the logistic rule was then constructed and an online risk calculator was generated.

**FIGURE 1:**
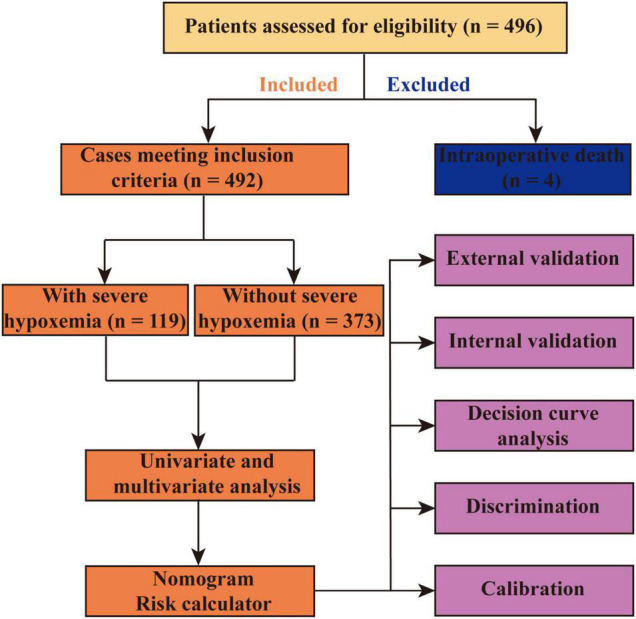
Flow chart of the study.

The development and internal validation (bootstrap method using 1000 replications) of the model were performed in the training set and the external validation was performed using data from the MIMIC-IV database. Both visual inspection and Hosmer–Lemeshow goodness-of-fit test were used to evaluate the calibration. The area under the receiver operating characteristic (ROC) curve (AUC) or c-index were used to assess the discrimination. The difference between two AUCs was compared by Delong method (11). Decision curve analysis was performed to assess the clinical utility. To balance important patient characteristics between groups, the propensity score matching analysis was performed with a 1:1 nearest neighbor matching algorithm (a caliper of 0.02) without replacement, and the propensity scores were calculated by the logistic regression model. The flow chart of this study is presented in Figure 1.

## Results

### Demographic Characteristics

Among the 496 eligible patients who underwent AADS, 4 died intraoperatively and were excluded. The remaining 492 patients met the inclusion criteria and were included in the present study (Figure 1). The mean age of the included patients was 49.6 ± 11.3 years and 75.6% of them were male patients. The overall morbidity rate of severe hypoxemia within the first 24 h after AADS was 24.2%, moderate hypoxemia was 46.1%, and 93.9% of the patients had a PaO_2_/FiO_2_ value of ≤300 mmHg.

Multiple underlying conditions and comorbidities existed in this study population. Hypertension was the most common comorbidity, existed in 68.1% of the patients, smoking history in 43.9%, drinking history in 35.8%, renal insufficiency in 35.2%, chronic bronchitis in 21.5%, cerebrovascular disease in 17.9%, peripheral vascular disease in 13.6%, gastrointestinal tract disease in 8.5%, cardiac surgery history in 6.5%, diabetes mellitus in 4.3%, and pulmonary artery hypertension in 2.8% of the patients.

### Development of the Risk Prediction Model

**FIGURE 2:**
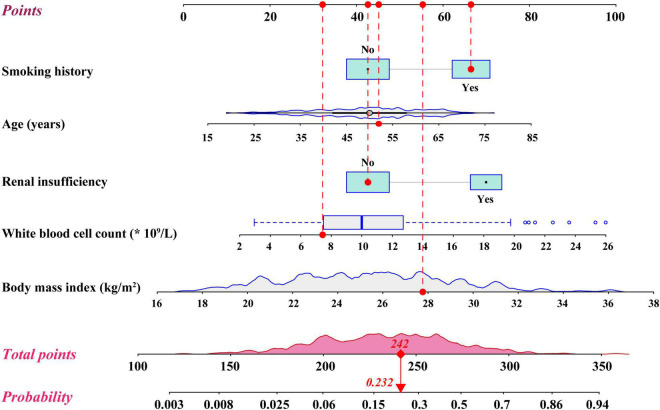
Nomogram for the prediction of severe hypoxemia in patients undergoing Stanford type A acute aortic dissection surgery.

The development of the risk prediction model for severe hypoxemia after AADS was conducted using data from our hospital. Firstly, we conducted univariate analysis to screen possible risk factors, the results of which are presented in Table 1. Collinearity diagnostics were conducted prior to the construction of a multivariate model. Factors with *P* < 0.1 or considered to be clinically important were further analyzed by multivariate logistic regression analysis. After screening, the variables that entered into the multivariate regression included male, age, body mass index, smoking history, drinking history, chronic bronchitis, pulmonary emphysema, peripheral vascular disease, renal insufficiency, diameter of the left atrium, white blood cell count, red blood cell count, hemoglobin, serum creatinine, combined surgical types, aortic root surgery, and the implantation of frozen elephant trunk. Multivariate analysis identified five significant risk factors in the final model, including older age, smoking history, renal insufficiency, higher body mass index, and white blood cell count (Table 2). A nomogram on the basis of these predictors and the logistic rule was then constructed to predict the probability of severe hypoxemia after AADS (Figure 2). The relative importance of these risk factors can be reflected by scaling their scores to 0–100 points based on the corresponding regression coefficients.

**TABLE 1:** Univariate analysis of possible risk factors for severe hypoxemia after AADS.

Characteristic	Without severe hypoxemia *n* = 373 (%)	With severe hypoxemia *n* = 119 (%)	χ^2^/*Z*/*t*	*P*-value
**Demographics**				
Male	266 (71.3)	106 (89.1)	15.434	<0.001
Age (years)	49.42 ± 11.27	50.35 ± 11.44	0.785	0.433
Body mass index (kg/m^2^)	24.71 ± 3.51	27.30 ± 3.60	6.968	<0.001
Smoking history	145 (38.9)	71 (59.7)	15.833	<0.001
Drinking history	124 (33.2)	52 (43.7)	4.291	0.038
**Underlying conditions**				
Hypertension	248 (66.5)	87 (73.1)	1.820	0.177
Diabetes mellitus	18 (4.8)	3 (2.5)	1.173	0.279
Chronic bronchitis	87 (23.3)	19 (16.0)	2.890	0.089
Pulmonary emphysema	20 (5.4)	4 (3.4)	0.778	0.378
Cerebrovascular disease	71 (19.0)	17 (14.3)	1.385	0.239
Peripheral vascular disease	58 (15.5)	9 (7.6)	4.892	0.027
Renal insufficiency	100 (26.8)	73 (61.3)	47.195	<0.001
Gastrointestinal tract disease	32 (8.6)	10 (8.4)	0.004	0.952
Atrial fibrillation	2 (0.5)	2 (1.7)	1.465	0.226
Cardiac surgery history	24 (6.4)	8 (6.7)	0.012	0.912
General surgery history	76 (20.4)	25 (21.0)	0.022	0.882
New York Heart Association III–IV	32 (8.6)	9 (7.6)	0.122	0.727
Pulmonary artery hypertension	13 (3.5)	1 (0.8)	2.283	0.131
Pericardial effusion	100 (26.8)	33 (27.7)	0.039	0.844
Diameter of the left atrium (cm)	3.5 (3.1, 3.8)	3.7 (3.4, 4.0)	3.861	<0.001
Diameter of the left ventricle (cm)	4.8 (4.4, 5.2)	4.9 (4.6, 5.3)	1.260	0.208
Diameter of the right atrium (cm)	3.7 (3.4, 4.0)	3.7 (3.5, 4.0)	0.906	0.365
Diameter of the right ventricle (cm)	3.6 (3.3, 3.8)	3.6 (3.4, 3.9)	1.378	0.168
Left ventricular ejection fraction (%)	62 (60, 65)	62 (60, 65)	0.398	0.691
**Laboratory values**				
White blood cell count (×10^9^/L)	9.5 (7.0, 12.0)	12.0 (9.2, 14.2)	5.682	<0.001
Red blood cell count (×10^12^/L)	4.2 (3.7, 4.5)	4.3 (3.9, 4.6)	2.556	0.011
Hemoglobin (g/L)	125 (113, 138)	133 (122, 141)	3.137	0.002
Platelet count (×10^9^/L)	159 (128, 207)	156 (120, 196)	1.059	0.290
Serum creatinine (μmol/L)	76.7 (63.9, 101.4)	98.3 (74.0, 136.7)	5.144	<0.001
Serum albumin (g/L)	37.9 (34.9, 40.9)	37.8 (34.9, 40.6)	0.328	0.743
Serum globulin (g/L)	25.6 (22.9, 28.5)	25.3 (22.5, 27.5)	1.158	0.247
**Operative variables**				
Surgical types			7.833	0.098
Isolated AADS	241 (64.6)	81 (68.1)		
Combined valve surgery	88 (23.6)	22 (18.5)		
Combined coronary surgery	21 (5.6)	5 (4.2)		
Combined valve and coronary surgery	16 (4.3)	11 (9.2)		
Combined other surgical types	7 (1.9)	0 (0.0)		
Aortic root surgery			1.964	0.580
Ascending aorta replacement	246 (66.0)	76 (63.9)		
David procedure	17 (4.5)	4 (3.4)		
Bentall procedure	94 (25.2)	36 (30.2)		
Other procedures	16 (4.3)	3 (2.5)		
Frozen elephant trunk	210 (56.3)	80 (67.2)	4.451	0.035

*AADS, Stanford type A acute aortic dissection surgery.*

**TABLE 2:** Multivariate analysis of independent risk factors for severe hypoxemia after AADS.

Characteristic	Coefficient	Standard error	OR (95% CI)	*P*-value
Renal insufficiency	1.010	0.243	2.746 (1.707–4.418)	<0.001
Smoking history	0.881	0.242	2.414 (1.503–3.879)	<0.001
Age (years)	0.039	0.012	1.040 (1.016–1.065)	0.001
Body mass index (kg/m^2^)	0.193	0.038	1.213 (1.125–1.307)	<0.001
White blood cell count (×10^9^/L)	0.130	0.033	1.139 (1.067–1.216)	<0.001

*AADS, Stanford type A acute aortic dissection surgery; CI, confidence interval; OR, odds ratio.*

By summing the points of all the predictors, the probability of severe hypoxemia in a postoperative patient can be easily predicted on the nomogram. Older patients who have smoking history, renal insufficiency, higher body mass index, and higher white blood cell count may have higher scores and resultant higher probabilities of severe hypoxemia. A concrete case is shown in Figure 2. To better accommodate the needs of modern clinical work, we also created and provided an interactive network risk calculator for severe hypoxemia after AADS which is available online.^3^

### Validation and Assessment of the Risk Prediction Model

**FIGURE 3:**
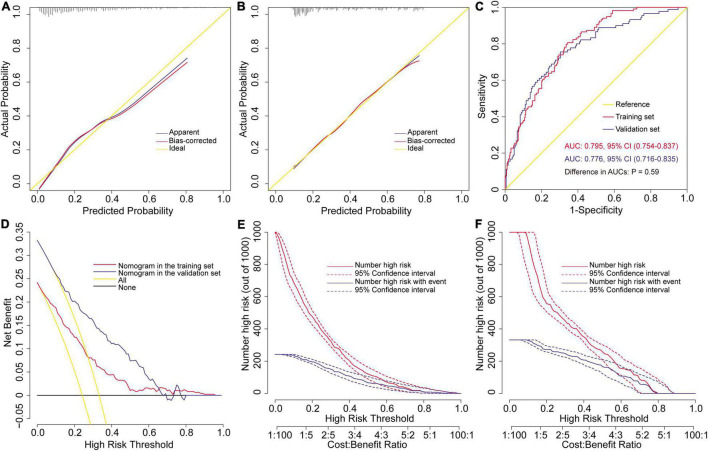
Assessment and validation of the preoperative nomogram for severe hypoxemia in patients undergoing Stanford type A acute aortic dissection surgery. Calibration plots in the training set **(A)** and the validation set **(B)**, ROC curves in the two sets **(C)**, decision curves in the two sets **(D)**, and clinical impact curves in the training set **(E)** and the validation set **(F)**. AUC, area under the receiver operating characteristic curve; CI, confidence interval; ROC, receiver operating characteristic curve.

The external validation of the risk prediction model for severe hypoxemia after AADS was conducted using data from MIMIC-IV database. After a series of screening, 272 eligible cases with complete medical records were finally extracted and further analyzed as a validation set. The model calibrated well in two datasets by both visual inspection and goodness-of-fit test, with Hosmer–Lemeshow χ^2^ values of 6.364 (*P* = 0.607, Figure 3A) in the training set and 3.271 (*P* = 0.916, Figure 3B) in the validation set. The model showed good discrimination in both the training set [AUC = 0.795, 95% CI, (0.754–0.837)] and the validation set [AUC = 0.776, 95% CI, (0.716–0.835)], without significant difference between the two AUCs (*P* = 0.594, Figure 3C). To assess the clinical utility of the model, we conducted decision curve analysis and plotted decision and clinical impact curves. The decision curves indicated that patients could obtain more clinical net benefits across almost the whole range of threshold probabilities than either the treat-all scheme and the treat-none scheme in both the training and validation sets (Figure 3D). The clinical impact curves also indicated that the model had remarkable clinical usefulness (Figures 3E,F).

We further investigated to identify independent risk factors for moderate and mild hypoxemia. The screening process was similar to that described above, which was also conducted using a univariate analysis and a multivariate logistic regression analysis. For moderate hypoxemia, in addition to the five independent risk factors identified above, hypertension was identified as another independent risk factors (Supplementary Table 1). The model showed good discriminative ability [AUC = 0.815, 95% CI, (0.770–0.859)] and calibrated well (Hosmer–Lemeshow χ^2^ = 4.668, *P* = 0.792). For mild hypoxemia, renal insufficiency was not significant by multivariate analysis and finally only four variables (age, smoking history, body mass index, and white blood cell count) remained in the logistic model (Supplementary Table 2). This model also showed excellent discrimination [AUC = 0.861, 95% CI, (0.795–0.927)] and calibration (Hosmer–Lemeshow χ ^2^ = 4.898, *P* = 0.768).

### Risk Stratification

Based on the prediction model for severe hypoxemia and clinical practice, we further performed a risk stratification to facilitate clinical application (Table 3). We selected predicted probabilities of 0.1 and 0.3 as the cutoff values and stratified the study population into 3 risk intervals named low, medium, and high risk groups, corresponding to points of <215, 215–251, and >251 on the graphical nomogram. In this study, approximately one-third of the patients were, respectively, divided into low (31.1%), medium (36.4%), and high risk groups (32.5%).

**TABLE 3:** Risk intervals of severe hypoxemia based on the nomogram and clinical practice.

Risk intervals	Low risk (<215 points)	Medium risk (215–251 points)	High risk (>251 points)
Estimated probability (%)	<10	10–30	>30
Observed probability, % (95% CI)	5.2 (4.8–5.6)	18.8 (18.0–19.7)	48.3 (46.0–50.7)
No. of patients (%)	153 (31.1)	179 (36.4)	160 (32.5)

*CI, confidence interval.*

### Clinical Outcomes

The overall mortality of the included patients was 9.96% (49/492), with a rate of 6.7% in patients without severe hypoxemia versus 20.2% in those with severe hypoxemia (*P* < 0.001). Significant differences of other poor outcomes were also observed between patients with and without severe hypoxemia in the results of univariate analysis (Table 4). To further reveal the relationship between severe hypoxemia and outcomes, we performed propensity score matching analysis and yielded 103 matched pairs of patients. In this new study population, the differences of mechanical ventilation, pneumonia, the length of ICU stay, the length of hospital stay and mortality remained significant between two groups. However, the statistical differences were eliminated concerning reintubation, tracheotomy and readmission to ICU, despite the absolute numbers and rates were higher in patients with severe hypoxemia (Table 5).

**TABLE 4:** Clinical outcomes in patients with and without severe hypoxemia after AADS.

Variables	All patients *n* = 492 (%)	Without severe hypoxemia *n* = 373 (%)	With severe hypoxemia *n* = 119 (%)	χ^2^/*Z*	*P*-value
Mechanical ventilation (h)	63.1 (40.3, 94.9)	57.1 (38.2, 86.8)	92.8 (63.1, 157.5)	7.389	<0.001
Pneumonia	170 (34.6)	102 (27.3)	68 (57.1)	35.421	<0.001
Reintubation	72 (14.6)	45 (12.1)	27 (22.7)	8.152	0.004
Tracheostomy	55 (11.2)	30 (8.0)	25 (21.0)	15.274	<0.001
Readmission to ICU	44 (8.9)	24 (6.4)	20 (16.8)	11.919	0.001
ICU stay (h)	154.3 (108.1, 254.5)	135.5 (91.0, 207.0)	230.2 (155.6, 368.6)	7.430	<0.001
Hospital stay (days)	21 (17, 27)	20 (16, 26)	23 (19, 33)	3.937	<0.001
Mortality	49 (10.0)	25 (6.7)	24 (20.2)	18.242	<0.001

*AADS, Stanford type A acute aortic dissection surgery; ICU, intensive care unit.*

**TABLE 5:** Clinical outcomes in patients with and without severe hypoxemia following AADS after propensity score matching.

Variables	Included patients *n* = 206 (%)	Without severe hypoxemia *n* = 103 (%)	With severe hypoxemia *n* = 103 (%)	χ^2^/*Z*	*P*-value
Mechanical ventilation (h)	70.0 (43.9, 120.0)	61.1 (40.5, 92.8)	91.2 (61.7, 141.3)	4.171	<0.001
Pneumonia	86 (41.7)	31 (30.1)	55 (53.4)	11.498	0.001
Reintubation	41 (19.9)	18 (17.5)	23 (22.3)	0.761	0.383
Tracheostomy	33 (16.0)	13 (12.6)	20 (19.4)	1.768	0.184
Readmission to ICU	25 (12.1)	9 (8.7)	16 (15.5)	2.231	0.135
ICU stay (h)	180.1 (114.3, 317.2)	144.0 (109.0, 275.1)	210.3 (154.5, 326.5)	4.049	<0.001
Hospital stay (days)	22 (18, 29)	21 (16, 27)	23 (19, 32)	2.134	0.033
Mortality	25 (12.1)	7 (6.8)	18 (17.5)	5.509	0.019

*AADS, Stanford type A acute aortic dissection surgery; ICU, intensive care unit.*

In the same way, we compared these outcomes between patients with and without moderate hypoxemia and mild hypoxemia. Compared to those without moderate hypoxemia, patients with moderate hypoxemia had significantly poorer outcomes in the results of univariate analysis (Supplementary Table 3). Nevertheless, the differences were eliminated concerning pneumonia, reintubation, readmission to ICU and the length of hospital stay after propensity score matching (Supplementary Table 4). The results of univariate analysis between patients with and without mild hypoxemia are displayed in Supplementary Table 5. After propensity score matching, no differences were observed with regard to those outcomes between patients with and without mild hypoxemia (Supplementary Table 6).

## Discussion

Hypoxemia has been recognized as an important indicator of increased risk of poor outcomes in patients undergoing cardiovascular surgery (11), which was again confirmed by the results of this study. The morbidity rate reported in the literature varied due to different definitions and surgical populations (3, 5–7). In this study, the observed incidence of severe hypoxemia after AADS was 24.2%, moderate hypoxemia was 46.1%, and 93.9% of the patients had a PaO_2_/FiO_2_ value of ≤300 mmHg. Adverse outcomes after AADS were more common than other cardiovascular surgeries due to the complicated procedures with greater trauma and longer surgical time. The overall mortality of this study population was 9.96%, similar to previous reports (1). However, the risk of mortality and several other poor outcomes increased significantly in patients suffering from severe and moderate hypoxemia, which highlighted the necessity of identifying significant predictors and constructing risk prediction models.

In this study, we used data from 492 patients who underwent AADS at a single cardiovascular center to identify independent risk factors and develop risk prediction models for severe, moderate, and mild hypoxemia. By univariate and multivariate logistic regression analysis, a total of six independent predictors related to the development of postoperative hypoxemia were identified, including age, body mass index, smoking history, hypertension, renal insufficiency, and white blood cell count. A nomogram predicting the probability of severe hypoxemia after AADS was then constructed and externally validated using data from MIMIC-IV database. The nomogram model preformed well with regard to discrimination, calibration, and clinical utility in both the training and validation sets. To our knowledge, this is the first attempt to construct and validate a nomogram and the first online risk calculator available in this field worldwide. No significant difference was found regarding the discriminative ability between the two sets, which minimized the possibility of overfitting and improved the reliability of generalization. Finally, three risk intervals were defined as low, medium, and high risk groups on the basis of the nomogram and clinical practice.

Older age has been reported to be associated with the development of hypoxemia in various surgeries (7, 12–14), which was consistent with the results of this study. Shi et al. investigated the incidence and risk factors for early postoperative hypoxemia in patients undergoing on-pump coronary artery bypass grafting (7). They found that the incidence rate of postoperative hypoxemia (PaO_2_/FiO_2_ ≤ 200 mmHg) within the first 24 h was 37.8% and older age was an independent predictor associated with early postoperative hypoxemia by multiple logistic regression analysis. Szeles et al. explored the risk factors for severe hypoxemia after myocardial revascularization, finding that age was an independent predictors of severe hypoxemia by multivariate analysis. In this study, the average age of the included patients was 49.6 years, which was younger than that of previous reports. Nevertheless, the incidence rate of postoperative hypoxemia increased significantly with age.

Another significant predictor identified by multivariate analysis for postoperative hypoxemia was body mass index, which contributed the widest range of weight among all these predictors. This was in agreement with the results of many previous studies, in which higher body mass index or obesity have been widely reported to be independently associated with the development of postoperative hypoxemia (5–8, 10, 13, 15, 16). Marco et al. conducted a single-center retrospective study in 5023 patients who underwent cardiac surgery to investigate the incidence and predictors of postoperative hypoxemia and its relationship with the length of ICU stay (5). They found that postoperative hypoxemia developed in 30.6% of the patients and obesity was an independent risk factor for postoperative hypoxemia, which was a determinant of the length of ICU stay. Another recent single-center study conducted by Gong et al. reported that the incidence of severe hypoxemia was 36.6% and increased body mass index was an independent predictors for severe hypoxemia in patients undergoing surgery for acute type A aortic dissection (6). They believed that the obvious decrease in lung compliance and respiratory resistance in obese patients may be associated with the breathing difficulties and resultant hypoxemia. In addition, inflammatory response and oxidative stress may be involved in the process of lung injury of aortic dissection caused by obesity, which provided new ideas for the treatment (6).

Smoking history as an independent predictor for postoperative hypoxemia has also been reported in various surgeries, which was also identified in our analysis (10, 17, 18). Santos et al. conducted a retrospective cohort study in patients undergoing coronary artery bypass graft to detect factors associated with the occurrence of hypoxemia and found that the incidence of hypoxemia was 55% and higher body mass index and smoking were significant predictors (10). Wetterslev et al. conducted a prospective study to explore the predictors for postoperative hypoxemia after upper abdominal surgery in patients without preoperative cardiopulmonary dysfunction (18). They found that PaO_2_ during anesthesia and an elaborated history of former smoking habits and pack-years were two independent risk factors which may provide new tools to select patients for further research in prevention of postoperative hypoxemia and complications. Another pilot cross-sectional study conducted by Mohamed et al. reported that smoking carried more risk of intraoperative deterioration of arterial oxygen tension compared to non-smokers during one lung ventilation for patients undergoing video-assisted thoracoscopic surgery and prediction of such intraoperative respiratory complication should be considered for such patients (17).

White blood cell count was also identified as independent risk factors for all the three categories of postoperative hypoxemia by multivariate analysis. As a biomarker reflecting systemic inflammatory response, the increase of white blood cell represents higher inflammatory responses which may contribute to respiratory dysfunction and thus relates to hypoxemia (19). Ge et al. conducted a single-center retrospective study to identify the independent risk factors for postoperative hypoxemia in patients with acute aortic dissection and developed and validated a nomogram model to facilitate the prediction (9). They found that white blood cell was independently associated with postoperative hypoxemia, with an OR value of 1.21 (95% CI: 1.06–1.40, *P* = 0.008). Another study designed to identify the risk factors for hypoxemia following surgical repair of acute type A aortic dissection by Liu et al. reported that hypoxemia occurred in 30% of the included patients and preoperative white blood cell count was independently related to the development of postoperative hypoxemia (20). Recently, the relationship between inflammatory response and hypoxemia has attracted increasing attention, which may provide new and enlightening insight (21).

Although not identified to be significant for the development of severe hypoxemia, hypertension was identified as an independent predictor for moderate hypoxemia in our analysis. This was very similar to the results of a previous study conducted by Zhou et al., in which they found that only hypertension was independent risk factors for postoperative hypoxemia (PaO_2_/FiO_2_ ≤ 200 mmHg) using logistic regression (3). However, when they evaluated the risk factors for severe hypoxemia (PaO_2_/FiO_2_ ≤ 100 mmHg), body mass index, preoperative white blood cell, and postoperative APACHE II score were significant by univariate analysis and body mass index and postoperative APACHE II score were independent risk factors by multivariate analysis. In addition, previous studies have identified that hypertension was associated with the development of postoperative headache, which may be also associated with postoperative hypoxemia due to the fact that hypoxemia may cause secondary headaches (22, 23). However, a retrospective study conducted to investigate the incidence, risk factors and outcomes of preoperative hypoxemia in patients with type A acute aortic dissection by Guo et al. reported that AAD patients treated with lower systolic blood pressure were more likely to have low oxygenation levels and systolic blood pressure was identified as an independent risk factor for preoperative hypoxemia (24). They believed that altered pulmonary circulation and insufficient tissue perfusion related to low blood pressure were responsible for the development of hypoxemia. In addition, they found that patients with preoperative hypoxemia had significantly higher mortality, longer intubation time, longer ICU stay, longer hospital stay, and lower daily living scale score.

Renal insufficiency was another independent predictors for both moderate and severe hypoxemia in our results. The relationship between renal insufficiency and respiratory system complications has been reported previously (25, 26). However, available studies focused on the association between hypoxemia and renal insufficiency are still limited. Zhou et al. reported that APACHE II score was independently associated with the development of severe postoperative hypoxemia, in which renal function may play an important role (3). Liu et al. reported that both postoperative relative hypoxemia and acute kidney injury were independent factors for endotracheal re-intubation following coronary artery bypass grafting, which may also reveal an inherent connection. The exact mechanism still needs further investigation, however, we speculate that inflammatory response and the regulation of erythropoietin production by the kidney and resultant oxygen delivery may play a role (27).

Several other preoperative predictors for postoperative hypoxemia have also been previously reported in the literature but were not identified to be significant by multivariate analysis in this study, such as female sex, low left ventricular ejection fraction, chronic obstructive pulmonary disease, and diabetes (6, 28–30). Some intraoperative variables have also been reported to be associated with the development of postoperative hypoxemia, such as surgical types and deep hypothermic circulatory arrest (9, 15, 20), however, these variables were also not significant in multivariate analysis in this study. Sheng et al. reported that the use of deep hypothermic circulatory arrest may increase the risk of postoperative hypoxemia to 11.6-fold (15), and Liu et al. reported that deep hypothermic circulatory arrest time >25 min may significantly also increase the risk of postoperative hypoxemia, with an OR value of 3.26 (95% CI, 1.18–8.99, *P* = 0.023) (20). However, the implantation of frozen elephant trunk did not significantly increase the risk of postoperative hypoxemia in multivariate analysis in this study. In addition, some studies included postoperative variables, such as blood transfusion, into multivariate analysis, and identified these factors as independent predictors in the final model (20), however, we did not include postoperative variables in our analysis. We did this primarily with the consideration that postoperative variables were not available early and this may affect the purpose of early prediction. Even so, our model performed and validated well in various aspects.

Our prediction models may make sense in individualized risk prediction, high-risk populations identification, and early prevention. Recent guidelines on mechanical ventilation in acute respiratory distress syndrome have provided evidence-based recommendations related to six interventions which may be also appropriate when applied to the high-risk populations identified by our risk models, including prone positioning, low tidal volume and inspiratory pressure ventilation, higher versus lower positive end-expiratory pressure, high-frequency oscillatory ventilation, lung recruitment maneuvers, and extracorporeal membrane oxygenation (31). Appropriate preventive interventions and specific treatment targeting high-risk populations identified by our model may achieve considerable economic benefits and better clinical outcomes.

There existed several limitations in this study. First, the data of the training set was retrospectively collected from a single cardiovascular center, which may limit the generalizability of the model due to the small sample size. However, we adapted appropriate statistical methods and externally validated the model in another independent dataset, which may reduce this weakness to some extent. Second, some possible predictors that may relate to the development of postoperative hypoxemia were not available in this study, such as preoperative oxygenation index and involved tracts of aortic dissection. Nonetheless, the established model using current predictors also demonstrated good calibration, discrimination and clinical utility. Third, the time frame in this study was within the first 24 h postoperatively and the values used to define the severity of hypoxemia was the worst one. The changes of PaO_2_/FiO_2_ with time was not analyzed. However, we believe that this practice fulfilled the purpose of our work and would not largely influence the quality of this study. Fourth, we only analyzed the relationship between hypoxemia and in-hospital outcomes and long-term follow-up after discharge was not performed, which should be strengthened in future work.

## Conclusion

Postoperative hypoxemia was prevalent in patients undergoing AADS, related to poor outcomes. This study first developed and externally validated a multivariate prediction model for severe hypoxemia after AADS using five independent predictors and conducted a nomogram and an online risk calculator. The model performed well with regard to calibration, discrimination and clinical usefulness. Three risk intervals were defined as low, medium, and high risk groups based on the nomogram and clinical practice. The model may have clinical utility in risk prevention and informed decision-making through individualized risk assessment and identification of high-risk patients. In addition, we identified six independent predictors for moderate hypoxemia and four independent predictors for mild hypoxemia in patients undergoing AADS, which also performed and fitted well.

## Data Availability Statement

The raw data supporting the conclusions of this article will be made available by the authors, without undue reservation.

## Ethics Statement

The studies involving human participants were reviewed and approved by the Ethics Committee of Tongji Medical College of Huazhong University of Science and Technology (IORG No. IORG0003571). Written informed consent for participation was not required for this study in accordance with the national legislation and the institutional requirements.

## Author Contributions

XD, XH, LW, JX, and PY: conception and design. XC, HW, and SW: administrative support. WS, SL, CT, and DW: provision of study materials or patients. DW, SL, WS, YS, and YD: collection and assembly of data. JW, JL, RL, and WS: data analysis and interpretation. All authors manuscript writing and final approval of manuscript.

## Conflict of Interest

The authors declare that the research was conducted in the absence of any commercial or financial relationships that could be construed as a potential conflict of interest.

## Publisher’s Note

All claims expressed in this article are solely those of the authors and do not necessarily represent those of their affiliated organizations, or those of the publisher, the editors and the reviewers. Any product that may be evaluated in this article, or claim that may be made by its manufacturer, is not guaranteed or endorsed by the publisher.

## Footnotes

1
https://mimic.mit.edu/

2
www.R-project.org/

3
https://hypoxemia-prediction.shinyapps.io/dynnomapp/

## Abbreviations

AADS: Stanford type A acute aortic dissection surgery
AUC: area under the receiver operating characteristic curve
CI: confidence interval
ICU: intensive care unit
OR: odds ratio
PaO_2_–FiO_2_: arterial oxygen tension–inspired oxygen concentration
ROC: receiver operating characteristic curve.
