# Supplementary material for: Preoperative Nomogram and Risk Calculator for Postoperative Hypoxemia and Related Clinical Outcomes Following Stanford Type A Acute Aortic Dissection Surgery
Source: Front Cardiovasc Med. 2022 Apr 25;9:851447. doi: 10.3389/fcvm.2022.851447 (PMC9082545; doi:10.3389/fcvm.2022.851447)
Supplement: Supplementary file 1 [file Data_Sheet_1.docx]

Supplementary table 1. Multivariate analysis of independent risk factors for moderate hypoxemia after AADS

| Characteristic | Coefficient | Standard error | OR (95% CI) | *P* value |
| --- | --- | --- | --- | --- |
| Age (years) | 0.030 | 0.011 | 1.030 (1.008-1.053) | 0.007 |
| Body mass index (kg/m2) | 0.147 | 0.035 | 1.158 (1.082-1.240) | <0.001 |
| Smoking history | 1.138 | 0.247 | 3.120 (1.922-5.066) | <0.001 |
| Hypertension | 1.124 | 0.242 | 3.076 (1.913-4.945) | <0.001 |
| Renal insufficiency | 1.070 | 0.298 | 2.916 (1.628-5.225) | <0.001 |
| White blood cell count (× 109/L) | 0.130 | 0.036 | 1.139 (1.061-1.222) | <0.001 |

AADS, Stanford type A acute aortic dissection surgery; CI, confidence interval; OR, odds ratio.

Supplementary table 2. Multivariate analysis of independent risk factors for mild hypoxemia after AADS

| Characteristic | Coefficient | Standard error | OR (95% CI) | *P* value |
| --- | --- | --- | --- | --- |
| Smoking history | 0.950 | 0.478 | 2.586 (1.013-6.599) | 0.047 |
| Age (years) | 0.039 | 0.017 | 1.040 (1.006-1.074) | 0.021 |
| Body mass index (kg/m2) | 0.354 | 0.078 | 1.425 (1.222-1.660) | <0.001 |
| White blood cell count (× 109/L) | 0.135 | 0.066 | 1.144 (1.006-1.301) | <0.040 |

AADS, Stanford type A acute aortic dissection surgery; CI, confidence interval; OR, odds ratio.

Supplementary table 3. Clinical outcomes in patients with and without moderate hypoxemia after AADS

| Variables | Without moderate hypoxemia  n = 346 (%) | With moderate hypoxemia  n = 146 (%) | χ^2^/Z | *P* value |
| --- | --- | --- | --- | --- |
| Mechanical ventilation (hours) | 45.8 (25.4, 78.9) | 66.5 (42.3, 111.2) | 5.796 | <0.001 |
| Pneumonia | 30 (20.5) | 140 (40.5) | 18.006 | <0.001 |
| Reintubation | 12 (8.2) | 60 (17.3) | 6.839 | 0.009 |
| Tracheostomy | 4 (2.7) | 51 (14.7) | 14.891 | <0.001 |
| Readmission to ICU | 6 (4.1) | 38 (11.0) | 5.956 | 0.015 |
| ICU stay (hours) | 112.3 (84.7, 158.5) | 171.4 (114.2, 302.7) | 7.492 | <0.001 |
| Hospital stay (days) | 19 (16, 25) | 22 (18, 28) | 3.364 | 0.001 |
| Mortality | 3 (2.1) | 46 (13.3) | 14.465 | <0.001 |

AADS, Stanford type A acute aortic dissection surgery; ICU, intensive care unit.

Supplementary table 4. Clinical outcomes in patients with and without moderate hypoxemia following AADS after propensity score matching

| Variables | Without moderate hypoxemia  n = 100 (%) | With moderate hypoxemia  n = 100 (%) | χ^2^/Z | *P* value |
| --- | --- | --- | --- | --- |
| Mechanical ventilation (hours) | 48.1 (35.6, 82.8) | 62.8 (40.7, 133.4) | 2.289 | 0.022 |
| Pneumonia | 25 (25) | 30 (30) | 0.627 | 0.428 |
| Reintubation | 10 (10) | 11 (11) | 0.053 | 0.818 |
| Tracheostomy | 3 (3) | 12 (12) | 5.838 | 0.016 |
| Readmission to ICU | 5 (5) | 12 (12) | 3.150 | 0.126 |
| ICU stay (hours) | 116.5 (86.9, 162.0) | 146.3 (107.8, 244.3) | 2.734 | 0.006 |
| Hospital stay (days) | 20 (16, 26) | 22 (16, 27) | 0.841 | 0.400 |
| Mortality | 2 (2) | 12 (12) | 7.680 | 0.006 |

AADS, Stanford type A acute aortic dissection surgery; ICU, intensive care unit.

Supplementary table 5. Clinical outcomes in patients with and without mild hypoxemia after AADS

| Variables | Without mild hypoxemia  n = 30 (%) | With mild hypoxemia  n = 462 (%) | χ^2^/Z | *P* value |
| --- | --- | --- | --- | --- |
| Mechanical ventilation (hours) | 42.3 (23.8, 70.2) | 63.5 (40.4, 104.4) | 2.920 | 0.003 |
| Pneumonia | 4 (13.3) | 166 (35.9) | 6.361 | 0.012 |
| Reintubation | 0 (0) | 72 (15.6) | 5.477 | 0.019 |
| Tracheostomy | 0 (0) | 55 (11.9) | 4.021 | 0.045 |
| Readmission to ICU | 0 (0) | 44 (9.5) | 3.138 | 0.076 |
| ICU stay (hours) | 106.8 (81.4, 142.6) | 156.4 (110.3, 259.9) | 4.276 | <0.001 |
| Hospital stay (days) | 20 (17, 22) | 22 (17, 27) | 1.613 | 0.107 |
| Mortality | 0 (0) | 49 (10.6) | 3.534 | 0.060 |

AADS, Stanford type A acute aortic dissection surgery; ICU, intensive care unit.

Supplementary table 6. Clinical outcomes in patients with and without mild hypoxemia following AADS after propensity score matching

| Variables | Without mild hypoxemia  n = 24 (%) | With mild hypoxemia  n = 24 (%) | χ^2^/Z | *P* value |
| --- | --- | --- | --- | --- |
| Mechanical ventilation (hours) | 44.7 (29.3, 79.8) | 41.7 (22.1, 65.9) | 0.536 | 0.592 |
| Pneumonia | 4 (16.7) | 2 (8.3) | 0.762 | 0.383 |
| Reintubation | 0 (0) | 0 (0) | - | - |
| Tracheostomy | 0 (0) | 0 (0) | - | - |
| Readmission to ICU | 0 (0) | 1 (4.2) | 1.021 | 0.312 |
| ICU stay (hours) | 110.5 (86.1, 152.3) | 112.9 (66.1, 139.1) | 0.052 | 0.959 |
| Hospital stay (days) | 19 (16, 22) | 20 (14, 26) | 0.041 | 0.967 |
| Mortality | 0 (0) | 0 (0) | - | - |

AADS, Stanford type A acute aortic dissection surgery; ICU, intensive care unit.
